# Study on the Enrichment Effect of *Suillus luteus* Polysaccharide on Intestinal Probiotics and the Immunomodulatory Activity

**DOI:** 10.3390/microorganisms14010004

**Published:** 2025-12-19

**Authors:** Hongfei Ji, Mei Li, Ruxue Wang, Decheng Mao, Zhuoyang Ji, Lizeng Peng, Wenjie Ding, Haiyu Ji

**Affiliations:** 1 School of Life Sciences, Yantai University, Yantai 264005, China; hongfei10052024@163.com (H.J.); lmei9206@163.com (M.L.); wangruxue1107@163.com (R.W.); decheng0313@163.com (D.M.); 15266266770@163.com (Z.J.); 2 Shandong Academy of Agricultural Science, Institute of Food and Nutrition Science and Technology, Jinan 250009, China; 3 College of Food Science and Engineering, Tianjin University of Science and Technology, Tianjin 300457, China; dingwenjie1120@163.com

**Keywords:** *S. luteus* polysaccharide, structural characteristics, gut microbiota, CD4^+^ T cell immunity

## Abstract

*Suillus luteus* is a highly prized edible fungus and demonstrates significant potential in the field of bioremediation, particularly for soil restoration and pollution mitigation. However, systematic research on the structural characteristics of the bioactive polysaccharides and regulatory effects on gut microbiota metabolism remains scarce. In this study, *S. luteus* polysaccharide (SLP) was obtained by hot water extraction and the structural characteristics were systematically determined, as well as the regulatory function on gut microbiota metabolism in a tumor-bearing mice model. Results showed that SLP exhibited an average molecular weight of approximately 1.90 × 10^6^ Da with Fuc:Man:Glc:Gal molar ratio of 0.37:1.00:0.72:0.54. The polysaccharide predominantly employed β-(1→4)-Man*p* as the backbone with α-(1→3)-Fuc*p*, α-(1→6)-Glc*p*, and α-(1→6)-Gal*p* as side chains. SLP administration of 200 mg/kg in tumor-bearing mice exerted enrichment effects of intestinal probiotics, including *Lactobacillus* and *Odoribacter*, which were associated with alterations in glyoxylate and dicarboxylic acid metabolism, ultimately enhancing CD4^+^ T cell immunity and resulting in a tumor suppression rate of 53.14%. This study provides a theoretical foundation and supporting data for the development of *S. luteus* polysaccharide in the functional food field.

## 1. Introduction

*Suillus luteus*, a widely distributed edible ectomycorrhizal fungus in China, possesses fruiting bodies rich in bioactive components such as polysaccharides, proteins, flavonoids, and ergosterol, and exhibits favorable flavor characteristics and potential physiological regulatory functions [1]. *S. luteus* demonstrates significant potential in ecological remediation. The inoculation effectively modulates soil microbial community structure, enhances nutrient accumulation, and reduces heavy metal content, finding practical application in soil improvement and pollution control [2,3]. However, the structural characteristics and functional properties of bioactive polysaccharides derived from *S. luteus* fruiting bodies have not been fully characterized, which limits the in-depth development and efficient utilization of *S. luteus.*

Fungal polysaccharides are macromolecular polymers that constitute one of the primary bioactive components of edible fungi [4]. Notably, the biological activity in vivo of polysaccharides does not stem from the direct killing of pathogens or tumor cells, and is closely associated with their unique structural characteristics, exhibiting a clear “structure-activity relationship”. Upon oral administration, they cannot readily penetrate the intestinal barrier into the bloodstream and thus cannot be absorbed intact by the body due to their macromolecular nature. Instead, their biological activity largely depends on metabolic regulation by the gut microbiota [5,6].

In recent years, with the advancement of microbiological research, the gut microbiota—recognized as the human “second genome”—and its close association with host health have emerged as a frontier in life sciences [7]. The collective metabolic capabilities encoded by its genomes far exceed those of the human genome itself, playing an irreplaceable role in physiological processes such as nutrient metabolism, immune development, and defense against pathogens. Tumor initiation and progression can induce intestinal dysbiosis, whilst the disrupted microbial structure further impairs the host’s immune surveillance function, creating a vicious cycle [8]. Therefore, nutritional intervention to target and modulate the gut microbiota structure and restore a healthy microecology, thereby stimulating the host’s anti-tumor immune response, has emerged as a highly promising novel strategy for cancer adjuvant therapy.

*S. luteus* exhibits extensive distribution and abundant resources across China, and the efficient extraction and functional characterization of its polysaccharide are crucial for resource conversion. This study employed hot water extraction followed by ethanol precipitation to obtain *S. luteus* polysaccharide (SLP) from the fruiting bodies, then the structure of SLP was systematically characterized, including the monosaccharide composition, molecular weight distribution, and functional group features. Further, animal experiments explored SLP’s immunomodulatory effects via gut microbiota metabolism. These findings would provide theoretical rationale and supporting data for developing *S. luteus* polysaccharides as functional food ingredients or pharmaceutical adjuvants.

## 2. Materials and Methods

### 2.1. Materials and Reagents

The following materials were obtained from respective suppliers: *S. luteus* fruiting bodies (Heilongjiang Lino Food Store, Harbin, China); anhydrous ethanol, sulfuric acid, and hydrochloric acid (Tianjin Jiangtian Chemical Technology Co., Ltd., Tianjin, China); monosaccharide standards and T-series dextran standards (T-3, T-10, T-70, T-100, T-500) (Beijing Solarbio Science & Technology Co., Ltd., Beijing, China) [9]; and monoclonal antibodies CD4-PE and CD8-FITC (ImmunoWay Bio-technology, San Jose, CA, USA). All other chemicals used were of analytical grade.

### 2.2. Animals and Cells

Kunming mice were supplied by Jinan Pengyue Laboratory Animal Breeding Co., Ltd. (Jinan, China). Mouse sarcoma S180 cells were acquired from the Shanghai Institute of Biological Sciences, Chinese Academy of Sciences.

### 2.3. Extraction of SLP

**Figure 1:**
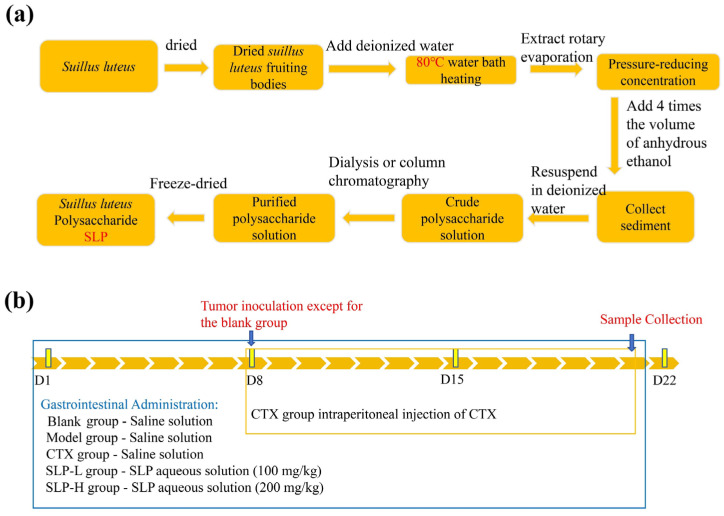
Preparation process (**a**) of SLP and the animal experimental design (**b**).

The preparation process for SLP is illustrated in Figure 1a. Dried *S. luteus* fruiting bodies were selected and placed in an 80 °C water bath at a solid-to-liquid ratio of 1:15–1:20 g/mL. The mixture was soaked and stirred for 3–4 h. The extract was subsequently subjected to concentration under reduced pressure using a rotary evaporator, then 4 times the volume of anhydrous ethanol was slowly added. The mixture was centrifuged at 4000 r/min for 15 min, and the precipitate at the bottom was collected. An appropriate amount of deionized water was added to resuspend the precipitants. This crude polysaccharide solution was purified using a dialysis bag with a molecular weight cut-off of 300 kDa, then freeze-dried for 24 h to yield the final *S. luteus* polysaccharide (SLP).

### 2.4. Chemical Composition Analysis of SLP

The polysaccharide content in SLP was quantified by the phenol-sulfuric acid method using glucose as a standard. Concurrently, the protein content was determined by the Coomassie Brilliant Blue colorimetric method with bovine serum albumin as the standard [9].

### 2.5. Average Molecular Weight and Monosaccharide Composition Analysis of SLP

SLP and T-series dextran standards aqueous solution of 1 mg/mL was prepared using deionized water and then filtered through 0.45 μm microporous membrane to remove impurities. Subsequently, the average molecular weight was determined using a high-performance gel permeation chromatography (HPGPC) system equipped with a TSK-gel G4000PWxL column (7.8 mm × 300 mm) [10].

Precisely 10 mg of dried SLP sample was weighed, 2 mol/L of trifluoroacetic acid solution was added, and this was hydrolyzed at 110 °C for 2 h. The hydrolysate was evaporated under reduced pressure to remove acid and redissolved in deionized water to yield the monosaccharides. Acetylation was performed by adding acetic anhydride and pyridine, followed by extraction with dichloromethane to obtain a solution of acetylated monosaccharide derivatives. Subsequently, the monomeric composition was determined by gas chromatography-mass spectrometry (GC-MS, 7000C/US1521U204, Agilent, Santa Clara, CA, USA) through an HP-5 capillary column, with derivative types identified through spectral library matching [11].

### 2.6. Detection of Primary Functional Groups in SLP

For Fourier Transform Infrared (FT-IR, Bruker VECTOR-22, Karlsruhe, Germany) spectroscopy, a total of 0.7 mg of lyophilized SLP was thoroughly mixed with 150 mg of dry KBr and ground to a homogeneous powder and then compressed into a transparent pellet. Subsequently, the spectrum was acquired in the region (4000–400 cm^−1^) to analyze the characteristic functional groups of SLP [12].

### 2.7. Micro-Morphological Observation of SLP

Freeze-dried SLP samples were adhered to conductive resin substrates, followed by uniform gold coating via gold sputtering. Prepared specimens were mounted on the scanning electron microscope stage and microstructural images were acquired at 200× magnification [13].

### 2.8. Glycosidic Bond Detection

The lyophilized SLP sample was dissolved in 0.5 mL of 99.9% D_2_O and the resulting solution, confirmed to be free of visible precipitates, was transferred into an NMR sample vial. NMR analysis was conducted using a 600 MHz DPX-600 spectrometer (Bruker, Ettlingen, Germany) including 5 spectra—^1^H-NMR, ^13^C-NMR, COSY, HSQC, and HMBC. Then the configuration of glycosidic bonds was determined through comprehensive analysis.

### 2.9. Animal Experiment Design

The animal experimental design is depicted in Figure 1b. Fifty male SPF-grade Kunming mice (25 ± 2 g) were housed under specific pathogen-free conditions with ad libitum access to feed and deionized water. The housing environment was maintained at 22–25 °C and 40–70% humidity, with a 12 h light/dark cycle. All procedures were approved by the Ethics Committee of Tianjin University of Science and Technology (Approval No.: 2023029, Date: 16 November 2023).

Following a one-week acclimatization, the mice were randomly divided into five groups (n = 10): blank, model, cyclophosphamide (CTX, 25 mg/kg/d), and SLP treatment groups (SLP-L, 100 mg/kg/d; SLP-H, 200 mg/kg/d) [14]. For the first 7 days, the SLP groups received oral gavage of the samples daily, while all other groups received equal volume of saline. On day 8, sarcoma S180 cells (2 × 10^6^ cells/mouse) were subcutaneously inoculated into the right axilla of all mice except the blank group. The gavage regimens continued for all groups until day 21, with the CTX group additionally receiving intraperitoneal injections of CTX [15].

### 2.10. Physiological Parameter Assessment

Upon termination of the experiment on day 22, body weights were recorded and solid tumors, spleens, and thymuses were collected and weighed. The relevant indices were calculated as follows: Organ Index (mg/g) = Immune Organ Weight (mg)/Body Weight (g).

Inhibition rate (%) = (1 − M_1_/M_2_) × 100%, where M_1_ and M_2_ are the mean tumor weights of the drug-treated and model groups, respectively [16].

### 2.11. 16S Amplicon Detection Method

The identification and quantification of gut microbiota were performed using the 16S rDNA amplicon sequencing method. Fecal samples were collected from the blank, model, CTX, and SLP-H groups. Genomic DNA was then extracted and assessed for quality by 1% agarose gel electrophoresis. The V3–V4 hypervariable regions of the 16S rRNA gene were amplified with barcoded primers (forward primer 341F, 5′-CCTACGGGNGGCWGCAG-3′; reverse primer 806R 5′-GGACTACHVGGGTW TCTAAT-3′), and the resulting PCR products were purified (2% agarose gel) and quantified using the QuantiFluor™-ST Blue Fluorescence Quantification System (Promega Corporation, Fitchburg, WI, USA). Subsequently, the quantified amplicons were ligated with Y-shaped adapters for library construction and underwent a second round of PCR amplification. Single-stranded DNA fragments were generated using the sodium hydroxide denaturation method. Following Illumina high-throughput sequencing, the obtained reads were compared with the NCBI Sequence Read Archive database [17].

### 2.12. Detection of Gut Microbiota Metabolites

Fecal samples from the model and SLP-H groups were pulverized in liquid nitrogen. Subsequently, 100 mg aliquots were homogenized in 500 μL of 80% methanol aqueous solution, incubated on ice for 5 min, and then centrifuged at 15,000× *g* for 20 min at 4 °C. The resulting supernatant was analyzed using a Dionex Ultimate 3000 HPLC system (Dionex Corporation, Chelmsford, MA, USA) equipped with a C18 column (2.1 mm × 100 mm, 1.7 μm). Data acquisition and processing were performed with Trace Finder 3.2.0 software for the qualification and quantification of small-molecule metabolites [18].

### 2.13. Methodology for Lymphocyte Subpopulation Analysis

Peripheral blood (50 μL) was collected from mice in each group. The samples were treated with 1 mL of red cell lysis buffer and incubated at room temperature for 10 min to remove erythrocytes. Subsequently, 2 μL of CD4-PE and CD8-FITC antibodies were added and mixed thoroughly and incubated for 20 min in the dark to allow the antibodies to bind specifically to CD4 and CD8 on lymphocyte surfaces. Following incubation, cells were washed with physiological saline to remove unbound antibodies. Then the cells were resuspended in 500 μL physiological saline, passed through a 300-mesh cell strainer, and subjected to quantitative analysis and data processing via a flow cytometry with CytExpert Version 2.5 software [19].

### 2.14. Data Analysis

All data are presented as the results of at least three independent replicates, expressed as mean ± standard deviation. Statistical analysis was performed by one-way ANOVA using SPSS 26.0 software. Differences were considered statistically significant at *p* < 0.05 and denoted in figures with “*” and “#” symbols. Data charts were derived from the original output files of the detection equipment or created using Microsoft Excel 2021 to visually illustrate trends and differences among groups.

## 3. Results

### 3.1. Preliminary Structural Analysis of SLP

**Figure 2:**
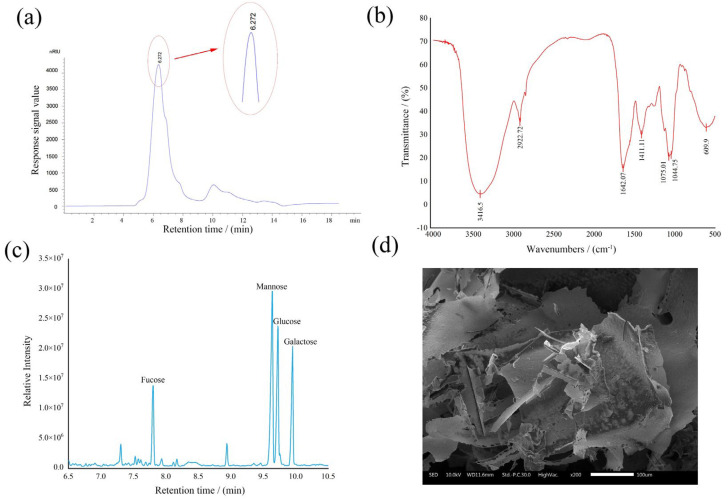
Preliminary structural characterization of SLP. (**a**) average molecular weight, (**b**) infrared spectrum, (**c**) monosaccharide composition results, (**d**) scanning electron micrograph 200× (**d**).

Chemical analysis revealed SLP’s polysaccharide contents to be 92.28 ± 3.76%, with protein contents at 4.92 ± 0.34%. The average molecular weight of SLP was determined with the results shown in Figure 2a. The chromatogram exhibited a single, narrow, and symmetrical signal peak, confirming SLP’s uniform molecular weight distribution and high purity after purification. Substituting the retention time of 6.272 min into the standard curve (y = −0.4601x + 9.1698, R^2^ = 0.9984, y denotes the logarithm of the molecular weight and x is the corresponding retention time) yielded an average molecular weight for SLP of approximately 1.90 × 10^6^ Da.

The characteristic functional groups of SLP were detected, and the results were presented in Figure 2b. Strong absorption peaks at 3416.5 cm^−1^ (O-H stretching vibration), 2922.72 cm^−1^ (stretching vibrations of methyl and methylene C-H bonds), and 1411.11 cm^−1^ (C-H bending vibration) represented the characteristic peaks of polysaccharides. The intense absorption peak at 1642.07 cm^−1^ was attributed to O-H bending vibrations, whilst the absorption peaks in the 1000–1200 cm^−1^ range were attributed to C-O-C glycosidic bonds within the polysaccharide [20].

As shown in Figure 2c, SLP was composed of four monosaccharides, fucose (Fuc), mannose (Man), glucose (Glc), and galactose (Gal), at a molar ratio of 0.37:1.00:0.72:0.54.

Figure 2d displays the microscopic morphology of SLP magnified 200-fold. SLP exhibits a dense, irregular lamellar structure with diverse morphologies. Some lamellae remain relatively intact with smoother surfaces, while others show fragmentation and delamination, yielding irregularly shaped fragments of varying sizes. Collectively, the lamellar structures interlock and interweave, exhibiting a complex microstructure. This provides intuitive microscopic evidence for subsequent investigations into the physicochemical properties and potential applications of this polysaccharide [21].

### 3.2. One-Dimensional Nuclear Magnetic Resonance Spectroscopy Analysis of SLP

**Figure 3:**
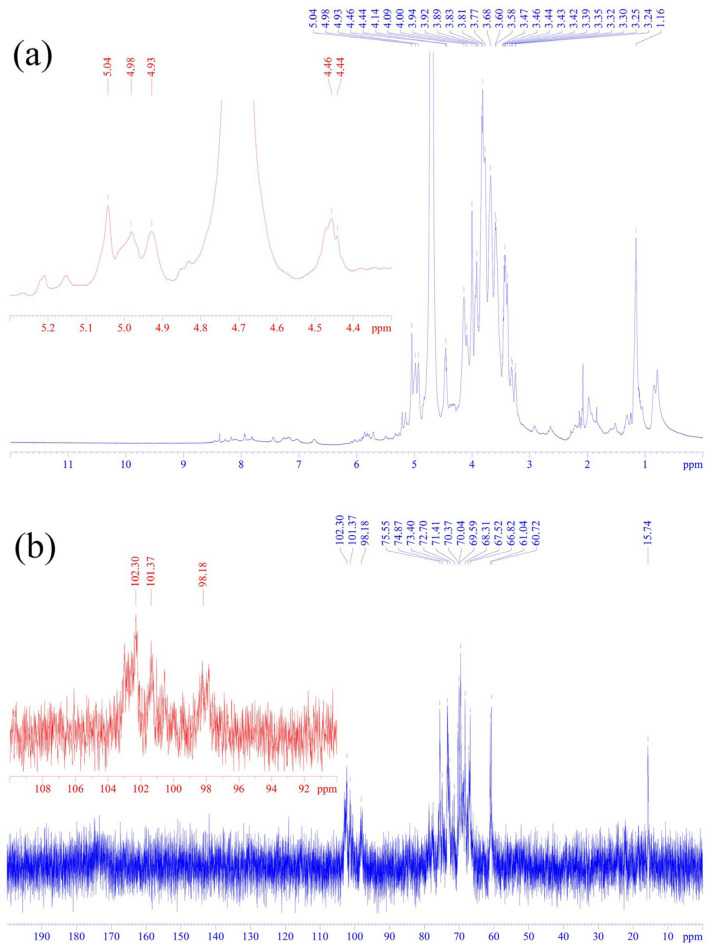
NMR ^1^H (**a**) and 13C spectra (**b**) of SLP.

Figure 3 presents the one-dimensional NMR spectra of the SLP. As shown in the ^1^H spectrum (Figure 3a), the characteristic anomeric proton signals were clearly identified. Specifically, the resonances observed at 5.02, 4.98, and 4.93 ppm were indicative of α-glycosidic linkages, while the peaks at 4.46 and 4.44 ppm were characteristics of β-glycosidic linkages. This co-existence of signals confirmed that SLP was a heteropolymer containing both α- and β-configurations. The complex set of signals in the region of 1.16 to 4.00 ppm was assigned to the ring protons (H2 to H6) of the glycosyl residues. The ^13^C spectrum (Figure 3b) provided corroborating evidence for the glycosidic linkage analysis. Key anomeric carbon signals were detected at 102.30, 101.37, and 98.18 ppm, which align with the anomeric proton signals in identifying the presence of both α- and β-configured sugar units. Furthermore, the signals for the remaining carbon atoms (C2 to C6) in the sugar rings were predominantly observed in the region of 60.72 to 75.55 ppm [22].

The 1D NMR spectra provided preliminary structural information for SLP. To comprehensively elucidate the types of monosaccharide residues and the linkages, 2D NMR techniques were employed for in-depth characterization.

### 3.3. Two-Dimensional NMR Spectral Analysis of SLP

**Figure 4:**
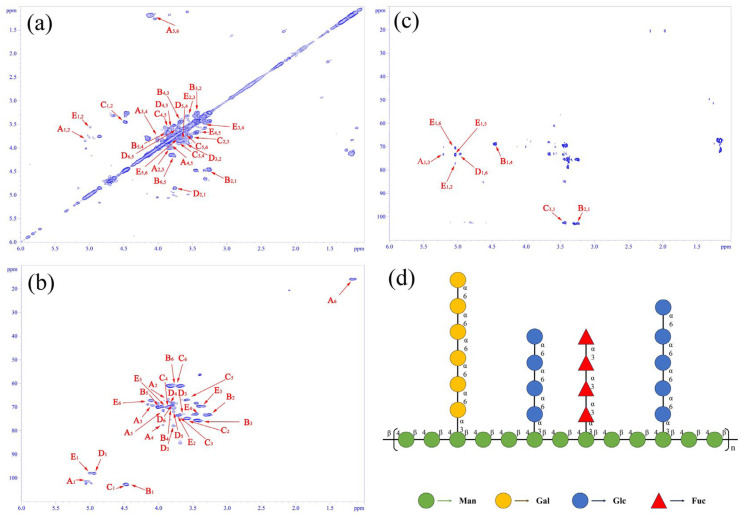
NMR COSY (**a**), HSQC (**b**), HMBC (**c**) and possible structural formula (**d**) of SLP.

Figure 4 presents the 2D NMR spectra of SLP. Figure 4a of COSY spectrum illustrates the correlation of adjacent hydrogen atoms in SLP with all labeled corresponding cross-absorption signals. The following monosaccharide residues were represented by single-letter codes: A for α-(1→3)-Fuc*p*, B for β-(1→4)-Man*p*, C for β-(1→3,4)-Man*p*, D for α-(1→6)-Glc*p*, and E for α-(1→6)-Gal*p*. The subscript numerals denote the sequence position of the sugar residue hydrogen atom, with the first digit representing the horizontal coordinate and the second the vertical coordinate.

Figure 4b analyzes the chemical shifts of C_1_ to C_6_ in fucose, mannose, glucose, and galactose residues in SLP. The chemical shift values of each carbon atom corresponded to the expected sugar residue types and glycosidic bond linkage patterns, providing a basis for determining the monosaccharide compositions and linkage positions.

Figure 4c identifies a complex series of cross-over signals, indicating that the SLP backbone consists of β-(1→4)-Man*p* and β-(1→3,4)-Man*p* residues, with β-(1→3,4)-Man*p* serving as the branching point. The side chains were composed of α-(1→3)-Fuc*p*, α-(1→6)-Glc*p*, and α-(1→6)-Gal*p* residues, linked to the corresponding carbon positions of the mannose residues in the backbone [23].

As depicted in Figure 4d, the probable structural formula of SLP was deduced based on the aforementioned results. The main chain consisted of randomly arranged β-(1→4)-Man*p* and β-(1→3,4)-Man*p* residues, with α-(1→3)-Fuc*p*, α-(1→6)-Glc*p*, and α-(1→6)-Gal*p* units linking to the carbon atoms of the backbone mannose as side chains [24].

In summary, SLP exhibited an average molecular weight of approximately 1.90 × 10^6^ Da, classifying it as a high-molecular-weight polysaccharide with monosaccharide compositions Fuc:Man:Glc:Gal of 0.37:1.00:0.72:0.54. Scanning electron microscopy revealed a lamellar structure where SLP molecules interlock and interweave, presenting a complex microstructure. Nuclear magnetic resonance analysis indicated that SLP was composed of β-(1→4)-Man*p* and β-(1→3,4)-Man*p* as the skeleton with α-(1→3)-Fuc*p*, α-(1→6)-Glc*p*, and α-(1→6)-Gal*p* serving as branches.

### 3.4. Effects of SLP on Physiological Parameters in Tumor-Bearing Mice

**Figure 5:**
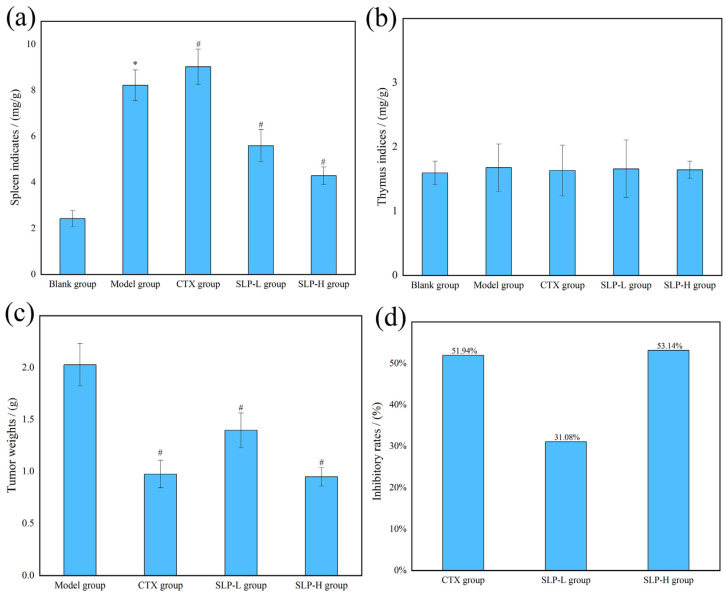
Effects of SLP on spleen indices (**a**), thymus indices (**b**), tumor weights (**c**) and inhibitory rates (**d**) in tumor-bearing mice. Note: *, *p* < 0.05, compared with blank group; #, *p* < 0.05, compared with model group.

The immunomodulatory effects of SLP on tumor-bearing mice were evaluated, and the key physiological parameters are shown in Figure 5.

Figure 5a,b presents the spleen and thymus indices, respectively. A significant increase (*p* < 0.05) in the spleen index was observed in the model group compared with the blank group, indicating that malignant tumor growth induced compensatory proliferation of immune organs. The spleen, being the largest peripheral immune organ, undergoes substantial activation and proliferation of T lymphocytes, B lymphocytes, and macrophages in an attempt to eliminate tumor cells, leading to increased spleen volume and weight [25]. SLP treatment at all doses tested significantly reduced the spleen index compared with the model group (*p* < 0.05), suggesting that SLP mitigated the immune organ damage caused by solid tumors and exerted certain protective effects. Figure 5b shows that there were no significant differences in thymus indices among the groups, indicating that the immunomodulatory functions of SLP may primarily act on the spleen rather than the thymus.

As demonstrated in Figure 5c,d, SLP intervention significantly inhibited the tumor’s growth. Tumor weights in all treatment groups were markedly lower than that in the model group (*p* < 0.05), leading to inhibition rates of 51.94%, 31.08%, and 53.14% for the CTX, SLP-L, and SLP-H groups, respectively. As a common chemotherapeutic agent, CTX inhibited transplanted tumor growth through the broad-spectra cytotoxicity, yet simultaneously caused non-selective damage to immune cells, leading to diminished immune functions [26], while SLP exhibited immune-activating properties by protecting immune organs, thereby effectively suppressing malignant proliferations of tumor cells. Notably, as a macromolecular polysaccharide that cannot directly enter the bloodstream to activate immune cells following oral administration, the immunomodulatory effects likely depended on interactions with the gut microbiota.

### 3.5. Effects of SLP on Gut Microbiota Diversity in Tumor-Bearing Mice

**Figure 6:**
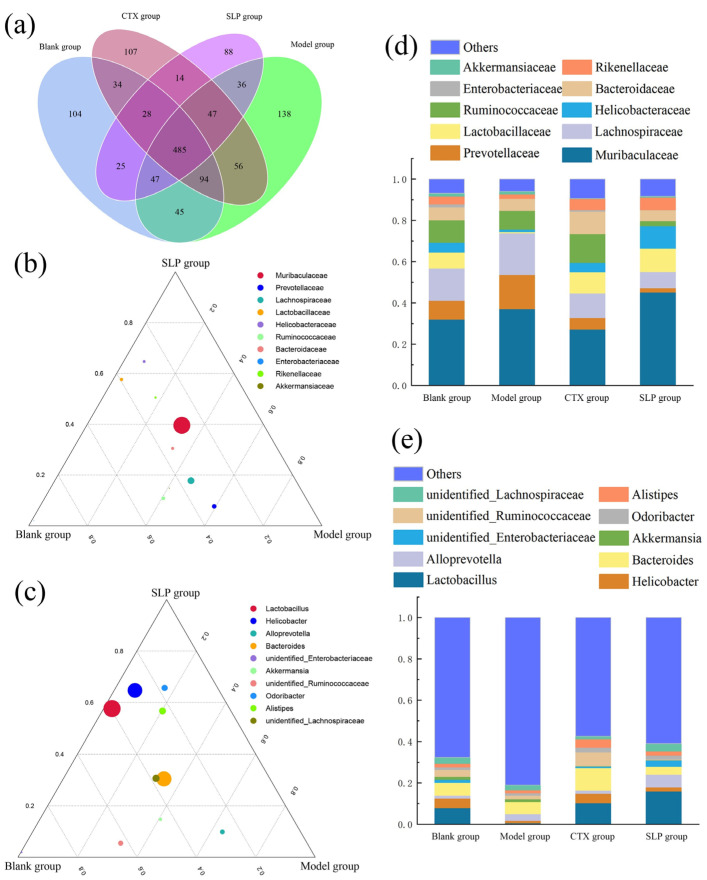
Effect of SLP on gut microbiota diversity in tumor-bearing mice. (**a**), Veen diagram; ternary phase diagram at family (**b**) and genus (**c**) levels; top 10 gut bacteria proportions at family (**d**,**e**) genus levels.

The gut microbiota play a pivotal role in regulating both anti-tumor and pro-tumor immune responses [27]. A gut microbiota diversity analysis was conducted by sequencing the V3–V4 region of 16S rRNA on fecal samples from each group, with results presented in Figure 6. Figure 6a shows a Venn diagram where each circle represents a group, and the numbers in overlapping and non-overlapping areas denote the counts of shared and unique operational taxonomic units (OTUs), respectively. As described, the four experimental groups collectively harbored 485 OTUs, with unique OTU counts of 104, 138, 107, and 88 for the blank, model, CTX, and SLP groups, respectively, indicating extensive gut microbiota diversity across experimental groups, potentially explaining the variations in anti-tumor immune responses.

Figure 6b,d presents a ternary phase diagram and column diagram of the top 10 gut bacteria at the family level in these groups. The three vertices in the ternary phase diagram represent the three sample groups, while circles denote species. Circle size is proportional to relative abundance, and proximity to a vertex indicates higher abundance of that species within the corresponding group. Compared with the model group, both the blank and SLP groups exhibited markedly increased relative abundances of Lactobacteraceae and Helicobacteraceae alongside a significant reduction in Prevotellaceae, suggesting a close association with the progression of solid tumors.

Figure 6c,e demonstrates intergroup differences in gut bacteria at the genus level. Compared with the model group, the SLP group exhibited a significantly elevated relative abundance of intestinal probiotics including *Lactobacillus*, *Helicobacter*, *Odoribacter*, and *Alistipes*. *Lactobacillus* is a well-recognized intestinal probiotic, and the increase in abundance can enhance the anti-tumor immune functions of the body through multiple metabolic pathways [28]. *Odoribacter* is a potential intestinal immune-enhancing symbiotic bacterium that plays a positive role in alleviating intestinal inflammation and maintaining the stability of the intestinal microecology [29]. *Alistipes* is a controversial genus with strain-dependent functions, showing bidirectional regulatory characteristics. Due to the current technical limitations, this study has not yet achieved precise identification of this genus at the species level. However, based on the overall experimental results, it is inferred that the *Alistipes* strains with increased abundance induced by SLP may belong to functional species with immunomodulatory activity [30]. *Helicobacter* is clearly identified as an immunosuppressive genus, and the increase in its abundance can weaken the anti-tumor immune response of the body. This phenomenon may be related to preventing autoimmune diseases caused by excessive immune activation [31]. However, the specific action mechanism still requires further investigation through integration with metabolomic data [32].

### 3.6. Effects of SLP on Gut Microbiota Metabolites in Tumor-Bearing Mice

Non-targeted metabolomics identified significantly differentially expressed metabolites between the model group and SLP group, as summarized in Table 1.

α-Ketoglutarate is a pivotal metabolite in the tricarboxylic acid cycle, participating in energy production and epigenetic regulation. Its downregulation may reverse the immunosuppressive microenvironment by inhibiting HIF-1α stability, impeding metabolic adaptation in tumor cells, and potentially promoting the differentiation of immune cells towards an anti-tumor phenotype [33]. Succinate, another vital TCA cycle intermediate, functions as a signaling molecule stabilizing HIF-1α and promoting inflammation. Its downregulation may diminish tumor-associated pro-inflammatory signaling, weaken tumor cell invasiveness, and aid in reprogramming macrophages from the pro-tumor M2 phenotype to the anti-tumor M1 phenotype [34]. Uridine, a key nucleotide for RNA synthesis, when downregulated, directly restricts tumor cell nucleotide pools, inhibiting nucleic acid synthesis essential for rapid proliferation. This indirectly enhances the competitive capacity of immune cells [6]. Multiple phosphatidylcholines and sphingomyelins constitute core structural components of cell membranes. Their extensive downregulation (e.g., PC (14:0/P-16:0), SM (d18:0/16:1(9Z)(OH))) indicates impaired membrane synthesis and stability in tumor cells, potentially heightening their susceptibility to cytotoxic T cell and NK cell-mediated killing [35]. Docosahexaenoic acid and eicosatetraenoic acid, crucial omega-3 polyunsaturated fatty acids, possess anti-inflammatory properties. Their downregulation may mitigate excessive anti-inflammatory environments, preventing immune cell functional suppression and favoring the maintenance of moderate immune activation [36]. FAHFA (18:2/18:1) represents a novel class of endogenous lipids exhibiting anti-diabetic and anti-inflammatory activity. Their downregulation may enhance T cell anti-tumor activity by reducing immune-suppressive signaling mediated by these compounds [37].

The 10-Nitrolinoleic acid ester serves as a definitive biomarker of oxidative stress. Its upregulation indicates significant oxidative/nitrosative stress within the tumor microenvironment. This stress state may induce tumor cell death or cause immune cell functional exhaustion, with its specific role dependent on stress intensity [38]. L-arginine is a conditionally essential amino acid and precursor for nitric oxide synthesis. Its upregulation may provide sufficient substrate for cytotoxic T cells and macrophages, promoting nitric oxide and polyamine synthesis. This is crucial for maintaining T cell proliferative activity and cytotoxic function [39]. N-methyl-D-aspartate (NMDA) is a neurotransmitter and agonist of NMDA receptors. Within immune cells, moderate NMDA receptor activation mediates calcium influx, enhancing T cell activation and cytotoxicity, thereby amplifying anti-tumor immune responses [40]. Upregulation of bile acid metabolites (e.g., 3-dehydrocholic acid) reflects alterations in the liver–gut axis. Gut microbiota-mediated changes in bile acid metabolism can directly regulate hepatic immune cell function via pathways such as foresaid X receptor, and may systemically influence anti-tumor immunity through the microbiota-immune axis [41]. However, the functions of some metabolites and their association with anti-tumor immunity remain unclear and require further experimental validation.

**Figure 7:**
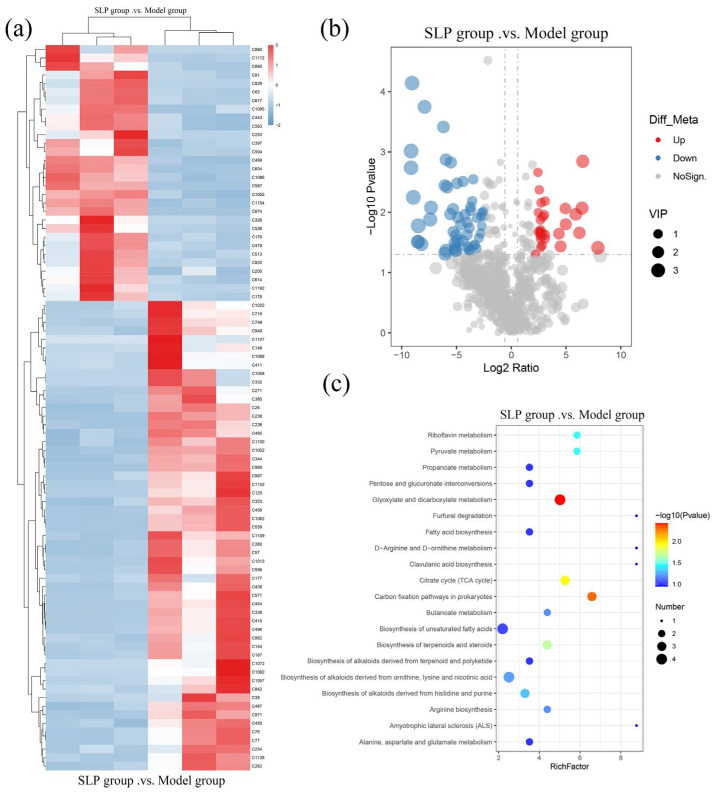
Effects of SLP on intestinal metabolites in hormonal mice. (**a**) joint hierarchical clustering of differential metabolites; (**b**) volcano diagram; (**c**) pathway-enriched bubble diagram.

Statistical and enrichment analysis of metabolites are summarized in Figure 7. Hierarchical clustering in Figure 7a reveals metabolite abundance patterns across samples, showing 85 significantly altered metabolites in the SLP group compared to the model group (*p* < 0.05), of which 55 were upregulated and 30 downregulated. Their functional implications in tumor-bearing mice warrant further investigation. The volcano plot (Figure 7b) corroborates these findings, with red and blue dots denoting significantly upregulated and downregulated metabolites, respectively. Finally, enrichment pathway analysis of these differentially expressed metabolites was performed using the KEGG database, with the results presented in Figure 7c. The bubble color gradient (blue → red) visually reflects the degree of pathway impact.

The results revealed two notably affected metabolic signaling pathways, the aldehyde and dicarboxylic acid metabolism pathway and the prokaryotic carbon fixation pathway, which may represent core targets for the SLP regulation of tumor-bearing mice metabolism and physiological functions. Carbon fixation refers to the biochemical process by which organisms convert inorganic carbon (mainly carbon dioxide) into organic carbon (such as sugars). This pathway has a relatively low relevance to the actual research content of this study. Its enrichment results may stem from indirect inferences in functional annotation or false positive signals in data analysis and thus no in-depth analysis was conducted.

Aldehyde and dicarboxylic acid metabolism broadly refer to metabolic processes involving dicarboxylic acids (such as malic acid and succinic acid), including their uptake, fermentation, and interconversion. Numerous gut bacteria excel at utilizing these organic acids as carbon and energy sources, and the upregulation of the glyoxylate and dicarboxylic acid metabolism pathway strongly indicates increased short-chain fatty acid (SCFA) production [42], revealing a fundamental metabolic reorganization occurring within the microbial community that favors SCFA synthesis [43].

Short-chain fatty acids (SCFAs), as the key signaling molecules of the gut microbiota, directly shape the differentiation and function of CD4^+^ T cells through mechanisms including epigenetic reprogramming, metabolic regulation, and cellular signal transduction [44]. On one hand, they robustly promote the differentiation of immunoregulatory T cells with immunosuppressive functions by inhibiting histone deacetylases and other mechanisms, thereby maintaining immune homeostasis and suppressing harmful inflammation. On the other hand, during immune response initiation, they empower effector CD4^+^ T cells by remodeling cellular metabolism and enhancing key gene transcription, thereby boosting cytokine production and long-term survival capacity, thereby amplifying immune offensive capabilities [45]. In summary, metabolites derived from the most fundamental energy metabolic pathways of gut microbiota directly construct a molecular bridge connecting microbial ecology and host immunity, providing profound theoretical support for intervening in immune-related diseases by targeting microbial metabolism.

### 3.7. Effects of SLP on T Cell Subset Distributions in Tumor-Bearing Mice

**Figure 8:**
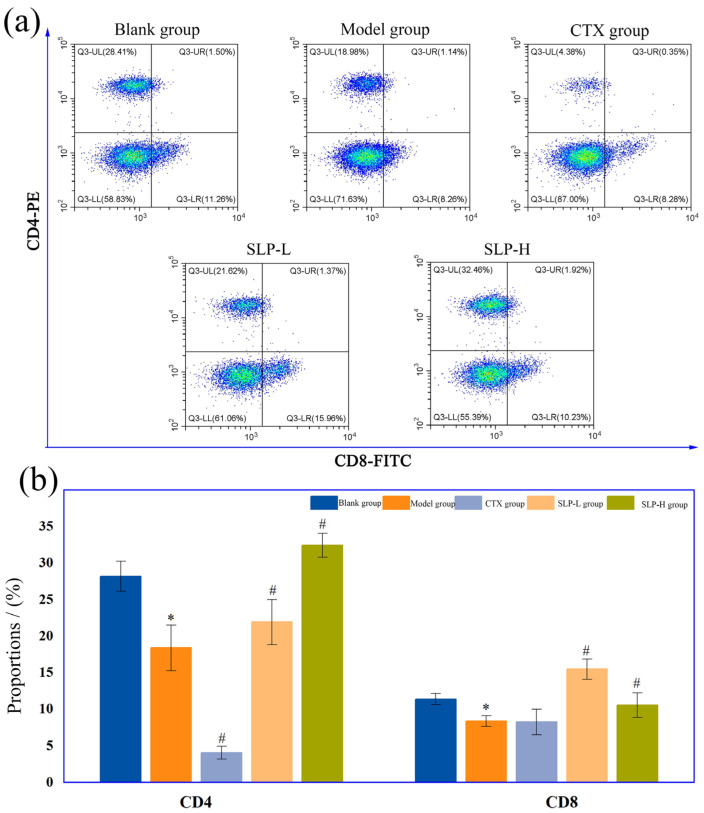
Effect of SLP on the distribution (**a**) and proportion (**b**) of T cell subpopulations in tumor-bearing mice. Note: *, *p* < 0.05, compared with blank group; #, *p* < 0.05, compared with model group.

The distributions and proportions of peripheral blood CD4^+^ and CD8^+^ T cells in the blank, model, CTX, and SLP groups were determined with the results presented in Figure 8. As depicted in Figure 8a, the CD4/CD8 double-stained scatter plots demonstrated excellent clustering, indicating the high reliability of the experimental results.

CD4^+^ T cells, being termed helper T cells, cannot directly kill infected cells or pathogens, as their primary functions are activating and assisting other immune cells by secreting cytokines and expressing surface molecules, thereby regulating the entire immune response [46]. In contrast, CD8^+^ T cells (cytotoxic T cells) are capable of directly killing abnormal target cells even with relatively weak antigen recognition ability.

As demonstrated in Figure 8b, compared with the blank group, the model group exhibited a marked reduction in CD4^+^ T cell proportions (*p* < 0.05), pointing to an impaired helper T cell response due to systemic immunosuppression. Correspondingly, the CD4^+^ T cell proportions in the SLP groups showed a marked recovery compared with the model group (*p* < 0.05), indicating that SLP activated the CD4^+^ T cells via the glyoxylate and dicarboxylic acid metabolic pathway and mediated SCFA production [47].

Regarding CD8^+^ T cells, the proportions remained relatively stable across groups. However, in the model and CTX groups, the ratio of CD8^+^ T cells decreased compared with the blank group, indicating that CTX, as a chemotherapeutic agent, exerted broad-spectrum inhibitory effects on both T cell types. In contrast, SLP exerted a more precise immunomodulatory effect by specifically enhancing CD4^+^ T cell functions and indirectly activating and recruiting CD8^+^ T cells. Consequently, the capacities of recognizing and eliminating tumor cells were remarkably enhanced [48]. However, the precise mechanism underlying metabolite-mediated activation of CD4^+^ T cells still requires further in-depth investigation.

## 4. Conclusions

In conclusion, the *S. luteus* polysaccharide (SLP) was extracted and purified, which presented average molecular weights of approximately 1.90 × 10^6^ Da with fucose, mannose, glucose, and galactose at a molar ratio of 0.37:1.00:0.72:0.54, and demonstrated a backbone of β-(1→4)-Man*p* and β-(1→3,4)-Man*p* with α-(1→3)-Fuc*p*, α-(1→6)-Glc*p*, and α-(1→6)-Gal*p* as side chains. In addition, high-dose (200 mg/kg) of SLP administration significantly modulated gut microbiota diversity in tumor-bearing mice, elevated the relative abundance of intestinal probiotics including *Lactobacillus* and *Odoribacter*, and mediated the metabolism of glyoxylate and dicarboxylic acids, which promoted SCFA production and ultimately regulated T cell immunity to control tumor growth in mice, achieving the inhibitory rate of 53.14%. In summary, this study provides a robust theoretical foundation and critical supporting data for the research and industrial application of SLP in the functional food field.

## Figures and Tables

**Table 1:** Differential metabolites in SLP-H group compared with model group.

ID	Description	Retention Time (min)	m/z	log2FC	*p*-Value	Up/Down
C1013	Uridine	0.58	243.06	−5.86	0.00	down
C1022	Xylobiose	7.49	281.09	−3.77	0.04	down
C1052	N-L-Acetyl-arginine	0.55	215.11	−5.14	0.04	down
C1057	1-linoleic acid glyceride	9.44	353.27	−4.96	0.00	down
C1058	α-ketoglutaric acid	17.47	145.01	−8.51	0.03	down
C1072	Cholesterol sulfate	10.22	465.30	−5.29	0.02	down
C1082	Glyceric acid	0.54	105.02	−9.05	0.00	down
C1088	Malic acid	0.57	133.01	−3.81	0.02	down
C1092	N-acetylgalactosamine	0.52	220.08	−6.06	0.00	down
C1100	Picolinic acid	0.59	122.02	−2.50	0.01	down
C1107	Succinic acid	10.86	117.02	−8.18	0.03	down
C1109	Tricarboxylic acid	16.93	175.02	−4.60	0.02	down
C1138	Anacardic acid	7.76	347.26	−4.24	0.01	down
C1155	LPG18:2	6.86	507.27	−6.20	0.00	down
C125	2,6-Dihydroxypurine	0.58	151.03	−9.15	0.00	down
C144	2-Hydroxybutanoicacid	0.93	103.04	−4.41	0.00	down
C149	2-Hydroxyvalericacid	0.62	117.06	−5.94	0.04	down
C177	3-Deoxy-lyxo-heptulosaricacid	7.97	221.03	−3.46	0.04	down
C187	3-Hydroxybutyricacid	0.60	103.04	−3.58	0.01	down
C226	4-Hydroxy-2-oxo-heptanedioate	0.57	189.04	−4.87	0.01	down
C238	4-Pyridoxicacid	0.78	182.05	−8.95	0.01	down
C254	5-oxo-3-phenyl-5-(2-quinolinylamino)pentanoic acid	3.53	335.14	−2.96	0.03	down
C26	(9R,10R)-Dihydroxyoctadecanoic acid	6.83	315.25	−3.13	0.01	down
C260	6-Deoxy-L-altrose	0.60	163.06	−5.10	0.04	down
C262	6-Keto-prostaglandinF1a	0.42	369.23	−4.00	0.04	down
C271	7-Methylxanthine	0.55	165.04	−5.97	0.00	down
C323	Adonitol	0.53	151.06	−3.34	0.03	down
C332	alpha-Ketoglutaric acid	0.55	145.01	−8.52	0.03	down
C338	Apiose	0.55	149.05	−7.32	0.01	down
C344	Behenic acid	11.83	339.33	−4.01	0.01	down
C38	1-(2,6-difluorobenzyl)piperidine hydrochloride	0.53	212.12	−3.66	0.04	down
C380	Cholic acid	4.72	407.28	−3.47	0.00	down
C410	D-(−)-Ribose	0.55	149.05	−7.40	0.01	down
C411	D-(+)-Malic acid	0.55	133.01	−4.03	0.02	down
C438	Dimethyl fumarate	0.58	143.03	−4.89	0.03	down
C450	DL-o-Tyrosine	2.87	182.08	−2.79	0.02	down
C454	Docosahexaenoic acid	8.55	327.23	−7.91	0.00	down
C456	Docosapentaenoic acid	8.93	329.25	−5.52	0.00	down
C465	Ecgonine	3.25	186.11	−2.68	0.02	down
C487	FAHFA(18:2/18:1)	8.84	559.47	−2.72	0.01	down
C496	Galactonic acid	0.57	195.05	−6.04	0.05	down
C556	Ketodeoxyoctonic acid	0.88	237.06	−5.04	0.02	down
C559	L-(−)-Glyceric acid	0.58	105.02	−9.14	0.00	down
C57	1,5-Anhydro-D-glucitol	0.55	163.06	−5.06	0.04	down
C571	L-glycero-D-manno-heptose	8.63	209.07	−5.16	0.03	down
C719	Neomentholglucuronide	6.24	331.18	−4.42	0.03	down
C749	Oxoadipic Acid	0.55	159.03	−5.56	0.01	down
C76	13,14-dihydro-15-ketoProstaglandinE2	8.14	351.22	−3.71	0.01	down
C77	13,14-Dihydro-15-keto-PGE2	9.25	351.22	−3.72	0.01	down
C842	PC(14:0/P-16:0)	13.13	690.54	−3.17	0.04	down
C862	PC(O-16:0/18:2(9Z,12Z))	0.52	744.59	−2.77	0.01	down
C949	SM(d18:0/16:1(9Z)(OH))	8.77	717.55	−2.56	0.01	down
C969	Stearic acid	9.94	283.26	−8.49	0.02	down
C971	Stercobilin	10.18	593.33	−5.31	0.01	down
C987	Tetrahydro-11-deoxycortisol	8.90	349.24	−3.85	0.04	down
C1055	ProstaglandinF1	7.60	355.25	2.68	0.02	up
C1095	N-methyl-D-aspartate	6.07	148.06	2.47	0.02	up
C1096	Oleinglyceride	10.11	355.29	2.44	0.00	up
C1112	VitaminB2	3.87	375.13	3.02	0.01	up
C1154	LPC20:5	5.32	540.31	4.50	0.04	up
C1192	N-morpholino-N-[(5-nitro-3-thienyl)carbonyl]urea	4.01	301.06	4.94	0.01	up
C176	3-dehydrocholicacid	6.73	407.28	3.09	0.02	up
C178	3-Hydroxy-11Z-octadecenoylcarnitine	7.15	442.35	2.71	0.02	up
C205	3-Oxo-5β-cholanate	8.00	373.27	2.21	0.05	up
C253	5-Methoxyindoleaceticacid	4.06	204.07	4.97	0.02	up
C326	AL8810Methylester	2.73	417.24	2.61	0.02	up
C397	Creatinine	0.57	114.07	2.47	0.01	up
C443	DL-Arginine	0.55	175.12	2.65	0.02	up
C479	ethyl5-hydroxy-4-oxo-4H-chromene-2-carboxylate	0.50	235.06	2.81	0.04	up
C489	FAHFA (2:0/21:0)	10.08	383.32	2.56	0.00	up
C504	Genipin	3.44	225.08	2.87	0.01	up
C513	Glycocholic acid	10.42	466.32	3.08	0.03	up
C526	HET0016	0.47	207.15	3.07	0.01	up
C563	L-Arginine	9.18	175.12	2.65	0.02	up
C567	Ethyllaurate	8.62	227.20	5.88	0.01	up
C604	LPC22:5	7.47	568.34	6.52	0.00	up
C614	LPE22:5	7.06	528.31	2.66	0.01	up
C63	10-Nitrolinoleate	7.12	326.23	2.68	0.03	up
C660	LysoPE(0:0/22:5(4Z,7Z,10Z,13Z,16Z))	6.25	528.31	2.73	0.01	up
C674	Myristic acid	8.60	227.20	6.46	0.01	up
C677	N-(3-Hydroxy-7-cis-tetradecenoyl)homoserinelactone	4.13	326.23	2.69	0.04	up
C825	PC (19:1/20:5)	7.98	820.59	4.35	0.02	up
C829	PC (20:3/20:4)	13.32	832.59	6.22	0.02	up
C860	PC (20:3(5Z,8Z,11Z)/20:3(5Z,8Z,11Z))	3.88	834.60	7.91	0.04	up
C91	1-Hexadecanoyl-2-(9Z-octadecenoyl)-sn-glycero-3-phospho-1-myo-inositol	13.21	837.55	2.87	0.02	up

## Data Availability

The original contributions presented in this study are included in the article. Further inquiries can be directed to the corresponding author.
